# 

**Published:** 1883-10

**Authors:** 


October, 1883.

NUMBER 4, VOL. XLVII.

THE

### CHICAGO MEDICAL JOURNAL & EXAMINER.

CHICAGO MEDICAL PRESS ASSOCIATION. AUG. 2D 1875.

CHICAGO:

*PUBLISHED BY THE CHICAGO MEDICAL PRESS ASSOCIATION*

188 CLARK STREET.

☞ **Read the Notice of this Journal among Advertisements.**

